# Coaxiality Evaluation of Coaxial Imaging System with Concentric Silicon–Glass Hybrid Lens for Thermal and Color Imaging

**DOI:** 10.3390/s20205753

**Published:** 2020-10-10

**Authors:** Tomoyuki Takahata

**Affiliations:** Graduate School of Information Science and Technology, The University of Tokyo, Tokyo 113-8656, Japan; takahata@jsk.imi.i.u-tokyo.ac.jp; Tel.: +81-3-5841-1669

**Keywords:** coaxial imaging system, silicon–glass hybrid lens, long-wavelength infrared camera, color image, thermal image

## Abstract

Thermal imaging is useful for tasks such as detecting the presence of humans and recognizing surrounding objects in the operation of several types of robots, including service robots and personal mobility robots, which assist humans. Because the number of pixels on a thermal imager is generally smaller than that on a color imager, thermal images are more useful when combined with color images, assuming that the correspondence between points in the images captured by the two sensors is known. In the literature, several types of coaxial imaging systems have been reported that can capture thermal and color images, simultaneously, from the same point of view with the same optical axis. Among them, a coaxial imaging system using a concentric silicon–glass hybrid lens was devised. Long-wavelength infrared and visible light was focused using the hybrid lens. The focused light was subsequently split using a silicon plate. Separate thermal and color images were then captured using thermal and color imagers, respectively. However, a coaxiality evaluation of the hybrid lens has not been shown. This report proposes an implementation and coaxiality evaluation for a compact coaxial imaging system incorporating the hybrid lens. The coaxiality of the system was experimentally demonstrated by estimating the intrinsic and extrinsic parameters of the thermal and color imagers and performing 2D mapping between the thermal images and color images.

## 1. Introduction

Thermal images are useful for various purposes, including for detecting the presence of humans by measuring body surface temperatures [1,2] and performing noninvasive measurement in agriculture [2,3,4]. Because the wavelength of long-wavelength infrared (LWIR) light captured by thermal cameras is approximately 10 times longer than that of visible light captured by conventional color cameras, thermal imagers generally have a smaller number of pixels than color imagers. Therefore, it is difficult to design and manufacture thermal-imaging cameras equipped with imaging sensors that incorporate large numbers of pixels. Combining thermal images with colored images effectively increases the amount of information captured. Thermal images can be useful in applications involving 3D mapping of thermal distributions in buildings [5,6] and object surface temperatures [7], combining color and depth information. Several researchers have reported the detection and tracking of humans using combinations of thermal and color images [8,9,10,11,12,13,14] or depth images [15]. Further, training datasets for machine learning algorithms for autonomous driving with thermal and color images [16] and with 3D light detection and ranging (LiDAR) data [17] have been presented. Thermal and color images can also be used for the semantic segmentation of transparent glass and pedestrians [18], for monitoring agricultural crops [19], and for improving visibility in the imaging of foggy environments [20].

The wavelength of the LWIR light used in thermal imaging lies in the range of 8–12 µm, which corresponds to the peak wavelength of light emitted by humans at or near room temperature (300 K) according to Planck’s radiation law. To date, there is no optical material that can be used to focus both visible and LWIR light. Glass and plastics used for manufacturing lenses to focus visible light cannot be used to focus LWIR light because they tend to absorb such light. Because germanium and silicon crystals are transparent to LWIR light, they are typically used as materials for producing LWIR lenses. However, they act as perfect reflectors for visible light.

Previous studies on methods of combining thermal and color cameras can be divided into two types: those in which the two cameras are placed side by side and those in which the two cameras are placed such that they detect perpendicular light from a 45° beam splitter.

In studies of the first type, two cameras (one each of the color and thermal types) were placed side by side [5,6,7,9,10,13,19]. Because the two cameras were operated with different optical axes in these studies, it was necessary to determine the lateral shift between the images captured by the two cameras to facilitate visible–LWIR image registration. However, the shift was observed to differ in accordance with the distances between the object and the two cameras, owing to the different optical axes. Because LWIR wavelengths are far longer than visible-light wavelengths, the thermal image of an object generally has a very different appearance with respect to its color image. Therefore, achieving accurate visible–LWIR image registration, using corresponding points or image features, is usually difficult.

In studies of the second type, thermal cameras have been designed to have the same optical axis as color cameras. This design, which can be called a coaxial system, is a rather ingenious method of easily combining thermal and color images. Studies based on the design of coaxial systems including thermal- and color-imaging cameras have been conducted previously [8,11,12,15,16,17,18]. In these studies, two cameras were combined using a hot mirror [8,11,12,15], which is a type of beam splitter that reflects LWIR light and transmits visible light, or a cold mirror [16,17,18,20,21], a beam splitter that reflects visible light and transmits LWIR light. Such a system ensures that the cameras operate on the same optical axis. Estimation of the intrinsic and extrinsic parameters of color and thermal cameras has been shown in the literature reports [20,21], in which Zhang’s algorithm [22,23] was used. However, because the beam splitter is placed in front of the camera lenses in this type of system, it must be large enough to span the entire viewing-angle range of the cameras. Fabrication of a compact coaxial system with the same design is difficult.

In a previous study, the authors presented a hybrid silicon–glass lens that consisted of a glass lens surrounded by a silicon Fresnel lens. This study experimentally demonstrated that LWIR and visible light from the same light source can be focused at the same point on a screen [24]. In another study, the authors designed an optical system to capture thermal and color images using a hybrid silicon–glass lens consisting of a plano-convex silicon lens and an achromatic glass lens [25]. The outer part of the lens was composed of silicon and focused on LWIR light to capture the thermal image, whereas the inner part was made of glass and focused visible light to capture the color image. Because the two lenses within the hybrid lens were aligned and glued before assembling the optical system, alignment of the LWIR and visible optical axes with the hybrid lens was easier than aligning separate cameras. However, an optimal optical system design has not been achieved, especially considering the sizes of both the color and thermal imagers, and methods of evaluating coaxiality have also not been shown.

This report presents a compact imaging system capable of capturing combined thermal and color images from the same viewing angle and optical axis, thanks to the use of a concentric silicon–glass hybrid lens. To ensure that the outer and inner lenses of the hybrid lens as well as the other optical elements were aligned well, the coaxiality of the imaging system was evaluated using thermal and color images capturing a point light source. The following two hypotheses regarding coaxial imaging systems were verified to evaluate the coaxiality of the prototype system.

**Hypothesis** **1.** *The thermal and color cameras have the same intrinsic and extrinsic parameters.*

**Hypothesis** **2.** *Using pairs of images of an object at a certain distance, mapping between the color and LWIR can be carried out, allowing automated visible–LWIR image registration for other distances.*

In the experiment, nine thermal and color image pairs were acquired of objects at each of three different distances, and thus a total of 27 image pairs were acquired. To verify Hypothesis 1, the intrinsic and extrinsic parameters of the thermal and color camera and the light source coordinates in each image pair were estimated using the 27 image pairs. The estimated parameters of the thermal and color cameras were compared with the designed parameters. To verify Hypothesis 2, mapping between the thermal and color images was estimated using nine pairs of images of a light source at a particular distance. The mapping was then evaluated by comparing the shift between a color image and a mapped thermal image using pairs of images of the light source at other distances. By verifying these hypotheses, it was experimentally demonstrated that the viewing points for both the thermal and color images were consistent with each another.

The remainder of this paper is organized as follows: Section 2 describes the design and implementation of the coaxial imaging system and methods of evaluating the coaxiality. Section 3 describes the results of capturing thermal and color images and estimating the parameters to verify the hypotheses. Section 4 discusses the relationship between the two hypotheses and directions for future work. Section 5 summarizes the study and states its conclusions.

## 2. Materials and Methods

### 2.1. Overview of Coaxial Imaging System

The hybrid lens described in [25] was used in this study. The lens was designed to have a focal length of approximately 50 mm when capturing both LWIR and visible light. The outer silicon lens, for LWIR light focusing, was 25.4 mm in diameter with a spherical surface, and a central hole with a diameter of 6.25 mm. The radius of curvature of the spherical surface was 126 mm. The inner lens, for capturing visible light, consisted of an achromatic doublet lens (#45-263, Edmund Optics Inc., Barrington, NJ, USA) with a diameter of 6.25 mm and a focal length of 50 mm. Both lenses were 3 mm thick.

**Figure 1:**
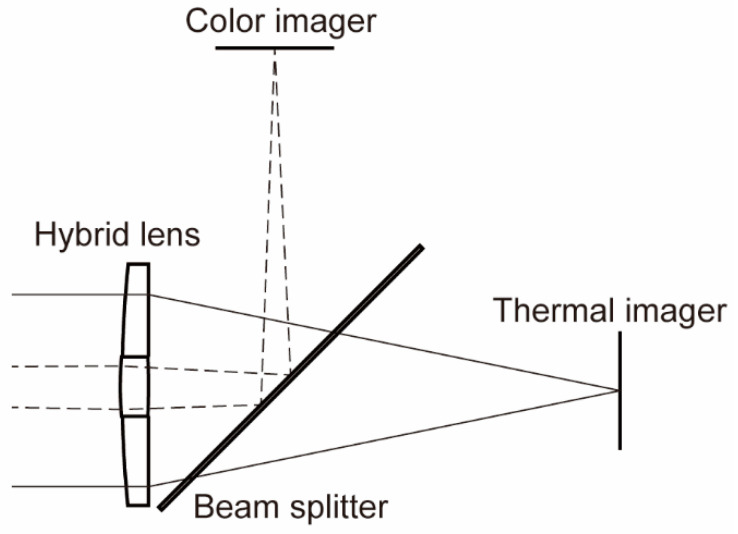
Schematic drawing of the proposed coaxial optical system.

Downstream from the lens, a beam splitter was used to split the incident light based on wavelength, to separate the LWIR and visible light, as shown in Figure 1. A 500 μm thick silicon wafer with a mirror-polished surface was used as the beam splitter. In general, a silicon plate transmits LWIR light, whereas a polished silicon surface serves as a mirror for reflecting visible light. After the beamsplitter, the LWIR and visible light were captured by the thermal and color imagers, respectively.

### 2.2. Design and Implementation

Because the hybrid lens was designed to have the same focal length for both LWIR and visible light, thermal and color imagers of the same size were desired to ensure the coaxial optical system possesses the same angle of view for the thermal and visible images. It is generally difficult to select imagers of the same size because the wavelength of LWIR light is an order of magnitude greater than that of visible light, and the pixel size of a thermal imager is typically larger than that of a color imager. In addition, color imagers have become increasingly small in recent years, owing to the miniaturization of modern cameras.

**Figure 2:**
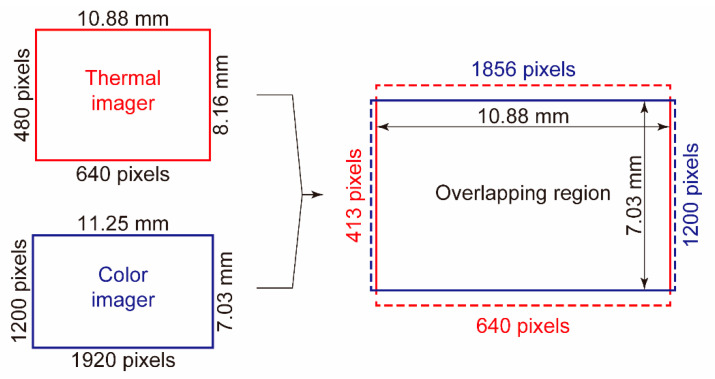
Overlapping region of thermal and visible imagers.

In this study, a relatively large color imager (IMX174LQ, Sony Corporation, Tokyo, Japan) with dimensions of 11.25 mm ×7.03 mm was used within a color camera (DFK 33UX174, The Imaging Source Asia Co., Ltd., Taipei City, Taiwan). The size of the color imager was nearly equal to that of the thermal imager (10.88 mm ×8.16 mm) used in the thermal camera (PI640, Optris GmbH, Berlin, Germany). Table 1 lists the specifications of the color and thermal cameras. Considering the small difference between the imager sizes, both the thermal and color images were captured in regions corresponding to the maximum possible overlap between the two rectangular image sensors, as shown in Figure 2. The number of overlapping pixels was 640×413 for the thermal images and 1856×1200 for the color images. The pixel count of the color imager exceeded that of the thermal imager by a factor of almost 8.4. The diagonal length of the overlapping region was 12.95 mm.

**Figure 3:**
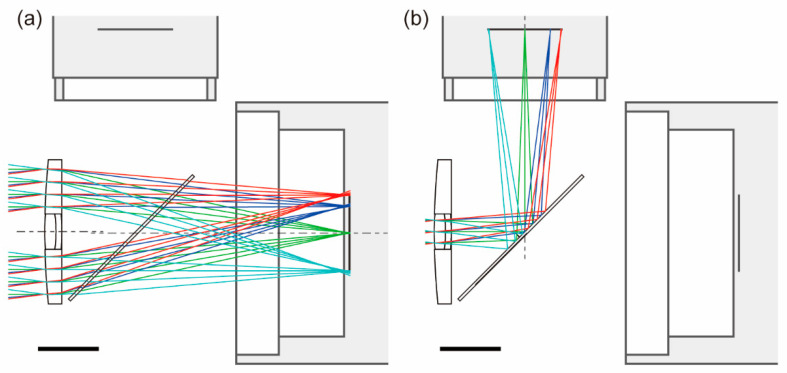
Results of ray-tracing simulation for hybrid silicon–glass lens and silicon beam splitter. (**a**) Long-wavelength infrared (LWIR) light focused using an outer silicon lens. (**b**) Visible light focused using an inner glass lens. The scale bars represent 10 mm.

The position and size of the beam splitter as well as the imager positions were determined by using ray-tracing simulation software (OSLO EDU edition, revision 6.6.5, Lambda Research Corporation, Littleton, MA, USA). Figure 3 presents the simulation results obtained for LWIR and visible light along with the camera cross-sections. Note that the color image must be horizontally inverted because visible light was reflected by the splitter. The viewing angle was 14.7°, as calculated based on the hybrid lens focal length of 50 mm and diagonal length of the overlapping region (12.95 mm).

**Figure 4:**
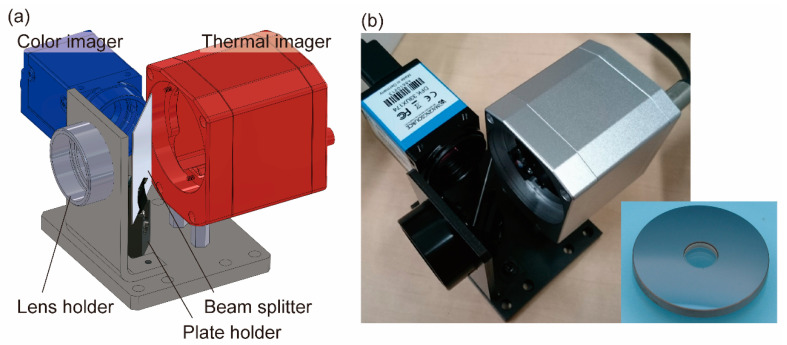
Design and implementation of a coaxial imaging system. A lens holder, plate holder, and thermal and color imagers were fixed on the same plate. A silicon beam splitter was placed in the plate holder. (**a**) Perspective view drawn with 3D CAD software (version 2017, SolidWorks, Dassault Systèmes SolidWorks Corporation, Waltham, MA, USA). (**b**) Photographs of the implemented system and silicon–glass hybrid lens.

A thermal camera (PI640) with its standard lens removed was used as the thermal imager, whereas a color camera (DFK 33UX174) was used as the color imager. The two imagers were placed at the positions determined via ray-tracing simulation. The beam splitter was placed on a plate holder (DH1, Thorlabs Inc., Newton, NJ, USA), and the plate holder was fixed on an aluminum plate (Figure 4a). The thermal and color cameras were fixed on the same plate using hexagonal spacers, and the heights of the cameras were adjusted with shim rings. Figure 4b provides a photograph of the actual imaging system employed in this study. The hybrid lens enables simultaneous adjustment of both thermal and color image planes via displacement of the lens along the optical axis. In this study, the focal plane was set at infinity, in the state shown in Figure 3.

### 2.3. Capturing Light Source Images

Both cameras were connected to a miniature computer (NUC 7i7BNH, Intel Corporation, Santa Clara, CA, USA). Color and thermal images were captured using Robot Operating System (ROS) packages uvc_camera [28] and optris_drivers [29], respectively.

To evaluate the coaxiality of the system, thermal and color pairs of images of an object that emitted light over a wide range of wavelengths from the LWIR to red (visible) wavelengths were captured. An infrared light source (IRS-001C, IR System Co., Ltd., Tokyo, Japan) was used as the object. The distance between the object and the front of the hybrid lens was set to 0.5, 1, and 2 m. At each distance, thermal and color images were captured for nine different positions of the object in the plane perpendicular to the optical axis. Thus, 27 image pairs were captured in total.

### 2.4. Camera Parameters for Thermal and Color Cameras

To verify Hypothesis 1, the intrinsic and extrinsic parameters of the color and thermal cameras were estimated. The parameters were defined based on a commonly used camera model without distortion.

A light source at a point Pw=[XYZ1]T in a world homogeneous coordinate system was mapped to a point Pc=[xyz1]T in a camera homogeneous coordinate system using an extrinsic parameter matrix [R|t], which combines a 3 × 3 rotation matrix R and translation vector t, as follows:

$$\left[\begin{array}{c} \begin{array}{c} x\\ y\\ z\\ 1 \end{array} \end{array}\right]=\left[\begin{array}{cc} R & t\\ 0 & 1 \end{array}\right]\;\left[\begin{array}{c} \begin{array}{c} X\\ Y\\ Z\\ 1 \end{array} \end{array}\right]=\left[\begin{array}{cccc} r_{11} & r_{12} & r_{13} & t_{x}\\ r_{21} & r_{22} & r_{23} & t_{y}\\ r_{31} & r_{32} & r_{33} & t_{z}\\ 0 & 0 & 0 & 1 \end{array}\right]\;\left[\begin{array}{c} \begin{array}{c} X\\ Y\\ Z\\ 1 \end{array} \end{array}\right]\;$$

Then, Pc was mapped to a point $p={\left[\begin{array}{cc} u & v \end{array}\right]}^{T}$ in an image coordinate system using the intrinsic parameter matrix A:

$$p=A\;\left[\begin{array}{c} x/z\\ y/z\\ 1 \end{array}\right]\;$$

A is defined by focal lengths in the *x*- and *y*-directions, $f_{x}$ and $f_{y}$, respectively, and the principal point $\left(c_{x},\;c_{y}\right)$:

$$A=\left[\begin{array}{ccc} f_{x} & 0 & c_{x}\\ 0 & f_{y} & c_{y} \end{array}\right]$$

In a commonly used model, the positions on the imagers, focal lengths, and principal points are normalized by the pixel size and have no units, and the origin for the principal point is set at the top left of the image. In this study, to eliminate the effect of pixel size and compare the design and experiment clearly, the positions on the imagers, focal lengths, and principal points were expressed in millimeters and the origin for the principal point was set at the center of the image. In other words, according to our model, for this design, $f_{x}=f_{y}=50$ mm and $c_{x}=c_{y}=0$ mm for both the thermal and color images.

**Figure 5:**
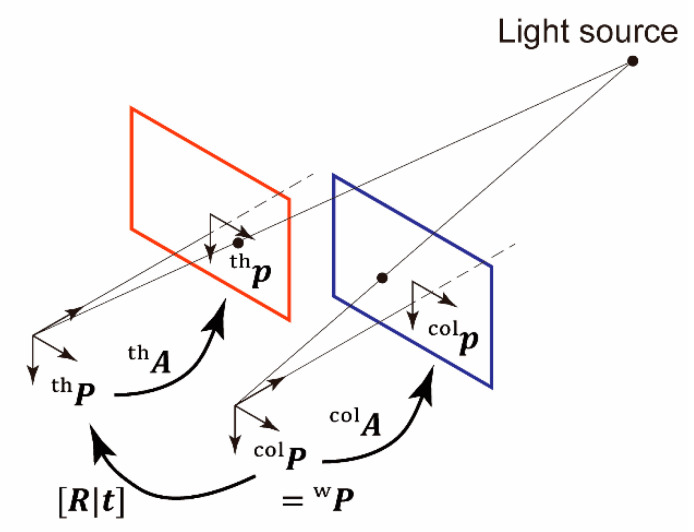
Relationships among world, thermal camera, color camera, thermal image, and color image coordinate systems.

In this study, the color camera coordinate system was assumed to be the same as the world coordinate system. Hence, Hypothesis 1 can be rephrased as follows: the extrinsic parameters of the thermal camera do not indicate rotation or translation, and the intrinsic parameter matrix of the thermal camera is the same as that of the color camera. Figure 5 illustrates the relationships among the coordinate systems.

For the color camera, a point Pw in the world homogeneous coordinate system is equal to a point Pcol in the camera homogeneous coordinate system as follows:

$$\left[\begin{array}{c} \begin{array}{c} x_{\mathrm{col}}\\ y_{\mathrm{col}}\\ z_{\mathrm{col}}\\ 1 \end{array} \end{array}\right]=\left[\begin{array}{c} \begin{array}{c} X\\ Y\\ Z\\ 1 \end{array} \end{array}\right]$$

Then, the point can be mapped to a point pcol in the color image coordinate system:

pcol=[ucolvcol]=[fcol,x0ccol,x0fcol,yccol,y] [xcol/zcolycol/zcol1]

However, for the thermal camera, a point Pth in the world homogeneous coordinate system can be mapped to a point in the camera homogeneous coordinate system:

$$\left[\begin{array}{c} \begin{array}{c} x_{\mathrm{th}}\\ y_{\mathrm{th}}\\ z_{\mathrm{th}}\\ 1 \end{array} \end{array}\right]=\left[R|t\right]\;\left[\begin{array}{c} \begin{array}{c} X\\ Y\\ Z\\ 1 \end{array} \end{array}\right]$$

Here, the rotation matrix can be expressed using a quaternion $q={\left[\begin{array}{c} \begin{array}{cccc} q_{x} & q_{y} & q_{z} & q_{w} \end{array} \end{array}\right]}^{T}$, which is normalized, i.e., |q|=1, and has three independent variables:

$$R=\left[\begin{array}{ccc} q_{x}^{2}-q_{y}^{2}-q_{z}^{2}+q_{w}^{2} & 2\left(q_{x}q_{y}-q_{z}q_{w}\right) & 2\left(q_{x}q_{z}+q_{y}q_{w}\right)\\ 2\left(q_{x}q_{y}+q_{z}q_{w}\right) & -q_{x}^{2}+q_{y}^{2}-q_{z}^{2}+q_{w}^{2} & 2\left(q_{y}q_{z}-q_{x}q_{w}\right)\\ 2\left(q_{x}q_{z}-q_{y}q_{w}\right) & 2\left(q_{y}q_{z}+q_{x}q_{w}\right) & -q_{x}^{2}-q_{y}^{2}+q_{z}^{2}+q_{w}^{2} \end{array}\right]$$

Then, the point can be mapped to a point pth in the thermal image coordinate system:

pth=[uthvth]=[fth,x0cth,x0fth,ycth,y] [xth/zthyth/zth1]

The intrinsic parameter matrices of the thermal and color cameras each have four variables, and the rotation matrix R and translation vector t each have three independent variables. When a pair of thermal and color images of the light source is captured, four equations are obtained for $u^{\mathrm{col}}$, $v^{\mathrm{col}}$, $u^{\mathrm{th}}$, and $v^{\mathrm{th}}$, and the coordinates of the light source are described by three variables, X, Y, and Z. Assuming that *n* pairs of images are obtained, the number of variables is 3n+10 and the number of equations is 4n. Therefore, when the number of image pairs *n* is >10, all the variables can be estimated.

The variables were estimated using the “scipy.optimize.minimize” function of scipy 1.4.1 run on Python 3.8.4. As arguments of this function, “method” was Sequential Least Squares Programming (SLSQP) [30,31] and “ftol” was $10^{-10}$. The variables to be estimated were the intrinsic parameters of the thermal camera $f_{\mathrm{th},x},\;f_{\mathrm{th},y},\;c_{\mathrm{th},x},\;\mathrm{and}\;c_{\mathrm{th},y}$; intrinsic parameters of the color camera $f_{\mathrm{col},x},\;f_{\mathrm{col},y},\;c_{\mathrm{col},x},\;\mathrm{and}\;c_{\mathrm{col},y}$; extrinsic parameters of the thermal camera q and t; and light source coordinates for each image pair in the world homogeneous coordinate system ${\left[\begin{array}{ccc} X_{i} & Y_{i} & Z_{i} \end{array}\right]}^{T}$. The objective function to be minimized through estimation was the weighted average of the root-mean-square errors (RMSEs) of the thermal and color images.

The initial values of the variables were set according to the design. The initial values of the intrinsic parameters of the thermal and color cameras were set as $f_{\mathrm{th},x}=f_{\mathrm{th},y}=f_{\mathrm{col},x}=f_{\mathrm{col},y}=50$ mm and $c_{\mathrm{th},x}=c_{\mathrm{th},y}=c_{\mathrm{col},x}=c_{\mathrm{col},y}=0$ mm. The initial values of the extrinsic parameters of the thermal camera were set to $q={\left[\begin{array}{c} \begin{array}{cccc} 0 & 0 & 0 & 1 \end{array} \end{array}\right]}^{T}$ and $t={\left[\begin{array}{ccc} 0 & 0 & 0 \end{array}\right]}^{T}$, indicating no rotation or translation. The initial values of the light source coordinates for each image pair were calculated as follows: the z-coordinate was set to the designed value (500, 1000, or 2000 mm) and the x- and y-coordinates were calculated using the coordinates on the corresponding color image, initial intrinsic parameters of the color camera, and z-coordinate.

The RMSEs of the thermal images were defined using the Euclidean distance between the i-th point on the captured thermal images $\left(\xi_{\mathrm{th},i},\;\eta_{\mathrm{th},i}\right)$ and the corresponding point mapped by the estimated parameters $\left(u_{\mathrm{th},i},\;v_{\mathrm{th},i}\right)$:

(1) $${\mathrm{RMSE}}_{\mathrm{th}}=\sqrt{\frac{1}{n}\overset{n}{\underset{i=1}{\sum }}\left[{\left(u_{\mathrm{th},i}-\xi_{\mathrm{th},i}\right)}^{2}+{\left(v_{\mathrm{th},i}-\eta_{\mathrm{th},i}\right)}^{2}\right]}\;$$

Here, n is the number of image pairs used for estimation and n=27 in this study. The coordinates on the captured images $\left(\xi_{\mathrm{th},i},\;\eta_{\mathrm{th},i}\right)$ were expressed in millimeters by scaling with the pixel size of the corresponding imager. The origin of the points was defined at the center of the image. The RMSE of the color images was defined as

(2) $${\mathrm{RMSE}}_{\mathrm{col}}=\sqrt{\frac{1}{n}\overset{n}{\underset{i=1}{\sum }}\left[{\left(u_{\mathrm{col},i}-\xi_{\mathrm{col},i}\right)}^{2}+{\left(v_{\mathrm{col},i}-\eta_{\mathrm{col},i}\right)}^{2}\right]}$$

Then, the objective function was defined as the weighted average of the two RMSE functions as

(3) $$\sqrt{\frac{w_{\mathrm{th}}}{w_{\mathrm{th}}+w_{\mathrm{col}}}{\left({\mathrm{RMSE}}_{\mathrm{th}}\right)}^{2}+\frac{w_{\mathrm{col}}}{w_{\mathrm{th}}+w_{\mathrm{col}}}{\left({\mathrm{RMSE}}_{\mathrm{col}}\right)}^{2}}$$

Here, weights for the thermal and color images $w_{\mathrm{th}}$ and $w_{\mathrm{col}}$ were used to make the errors in the color images smaller than those of the thermal images, taking advantage of the high resolution of the color images. In the estimation described below, the weights were set to 1/5.86 and 1/17.0, the reciprocals of the pixel sizes of the color and thermal imagers, respectively.

### 2.5. Mapping from Thermal to Color Images

To verify Hypothesis 2, homogeneous coordinate mapping from the thermal to the color images was estimated. The mapping of a point $\left(\xi_{\mathrm{th}},\;\eta_{\mathrm{th}}\right)$ in the thermal image coordinate system to a point $\left(\xi {’}_{\mathrm{th}},\;\eta {’}_{\mathrm{th}}\right)$ can be expressed using a rotation angle θ, translation vector ${\left[\begin{array}{cc} a & b \end{array}\right]}^{T}$, and scaling factor s as follows:

$$\left[\begin{array}{c} \xi {’}_{\mathrm{th}}\\ \eta {’}_{\mathrm{th}}\\ s \end{array}\right]=s\left[\begin{array}{ccc} \cos\theta & -\sin\theta & a\\ \sin\theta & \cos\theta & b\\ 0 & 0 & 1 \end{array}\right]\left[\begin{array}{c} \xi_{\mathrm{th}}\\ \eta_{\mathrm{th}}\\ 1 \end{array}\right]$$

**Figure 6:**
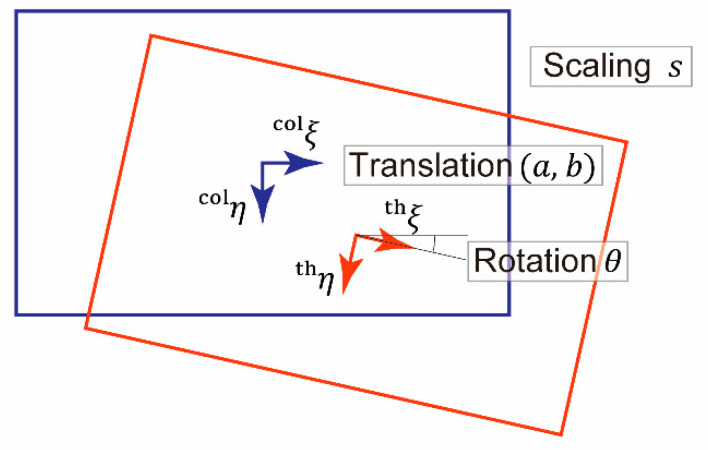
Mapping from thermal image to color image.

The mapping is depicted in Figure 6.

The above-mentioned four variables, θ, a, b, and s, were estimated using Python code via the function and arguments described in Section 2.4. The variables were estimated with the thermal and color image pairs capturing the light source placed at a specific distance from the imaging system (0.5, 1, or 2 m). The initial values of the parameters were set as follows: θ=a=b=0 and s=1.

The objective function to be minimized through estimation was the root mean square of the Euclidean distance between the i-th point on the color image $\left(\xi_{\mathrm{col},i},\;\eta_{\mathrm{col},i}\right)$ and the mapped point $\left(\xi {’}_{\mathrm{th},i},\;\eta {’}_{\mathrm{th},i}\right)$ in the corresponding thermal image:

(4) $$\sqrt{\frac{1}{N}\overset{N}{\underset{i=1}{\sum }}\left[{\left(\xi {’}_{\mathrm{th},i}-\xi_{\mathrm{col},i}\right)}^{2}+{\left(\eta {’}_{\mathrm{th},i}-\eta_{\mathrm{col},i}\right)}^{2}\right]}\;.$$

Here, N is the number of image pairs used for estimation and a value of N=9 was used in this study. Then, utilizing the images of the light source at the other distances, the estimated mapping was evaluated via the same objective function (Equation (4)).

## 3. Results

### 3.1. Captured Thermal and Color Images

**Figure 7:**
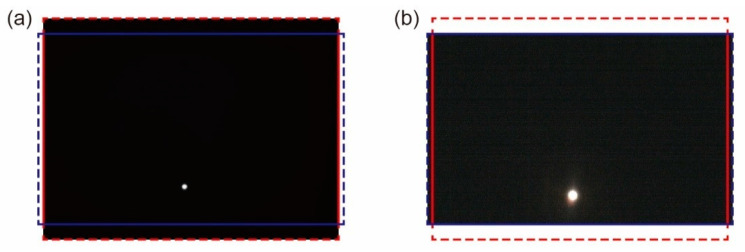
Example of a pair of captured images of a light source. (**a**) Thermal image and (**b**) color image (image pair No. 7 in Figure A1). The red and blue frames are identical to those shown in Figure 2.

**Figure 8:**
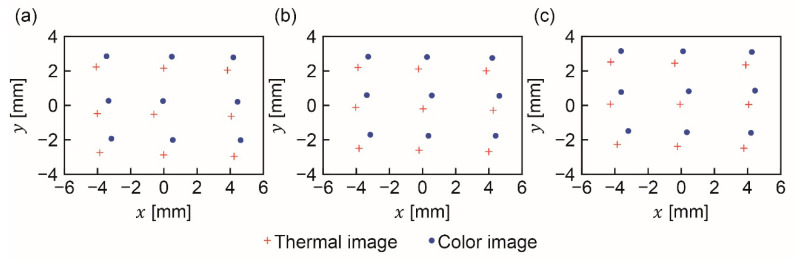
Positions of captured light sources at distances of (**a**) 0.5, (**b**) 1, and (**c**) 2 m on thermal and color images.

**Figure A1:**
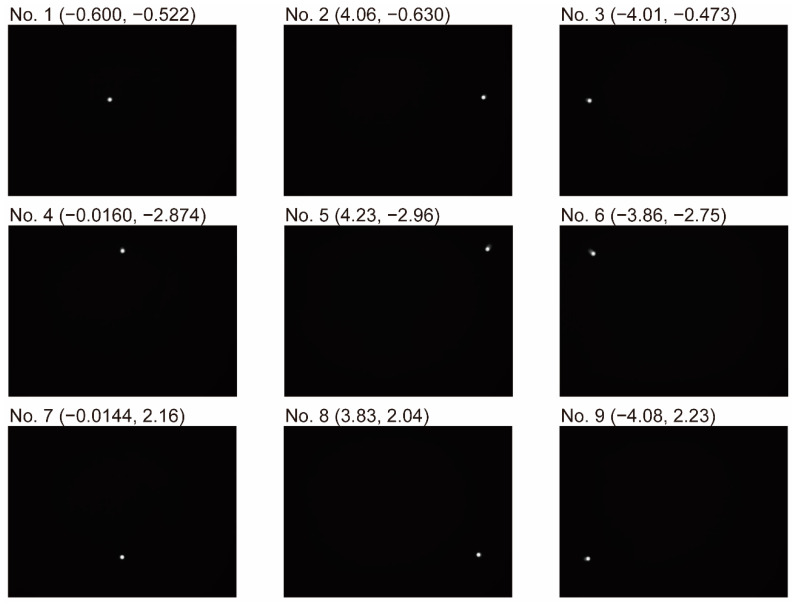
Captured thermal images of the light source at 0.5 m from the hybrid lens.

**Figure A2:**
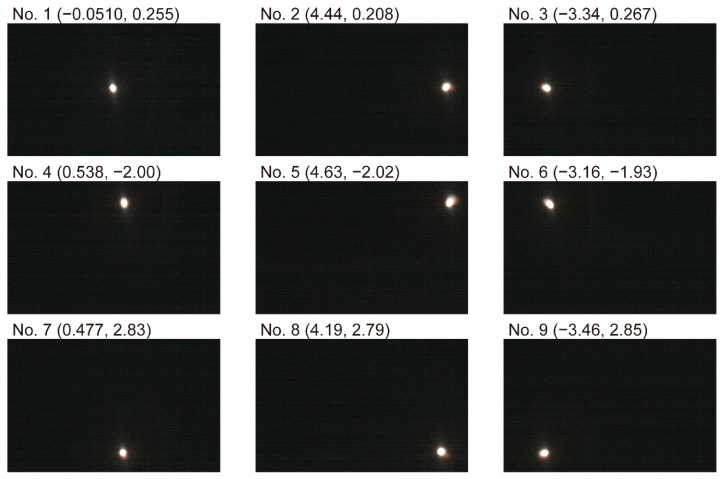
Captured color images of the light source at 0.5 m from the hybrid lens.

**Figure A3:**
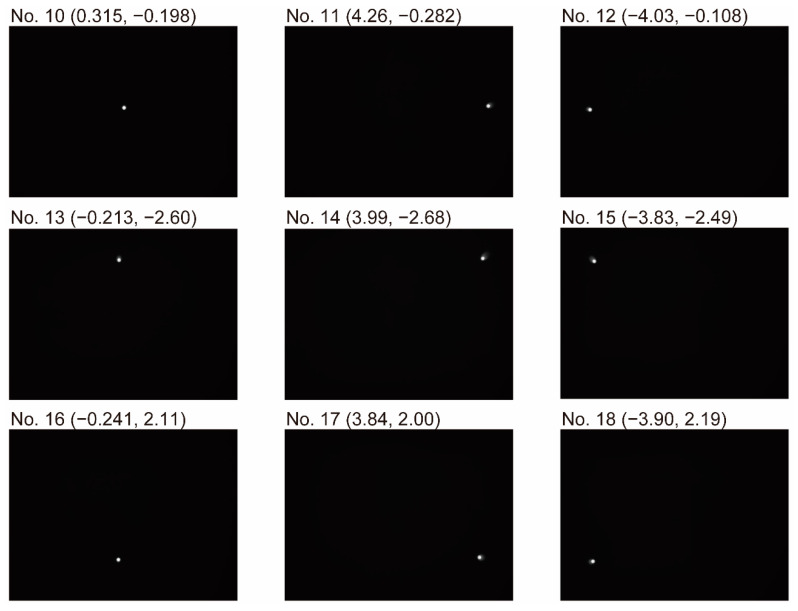
Captured thermal images of the light source at 1 m from the hybrid lens.

**Figure A4:**
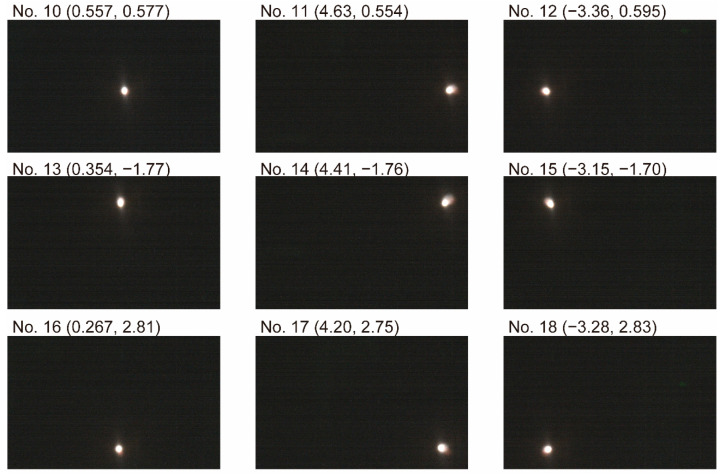
Captured color images of the light source at 1 m from the hybrid lens.

**Figure A5:**
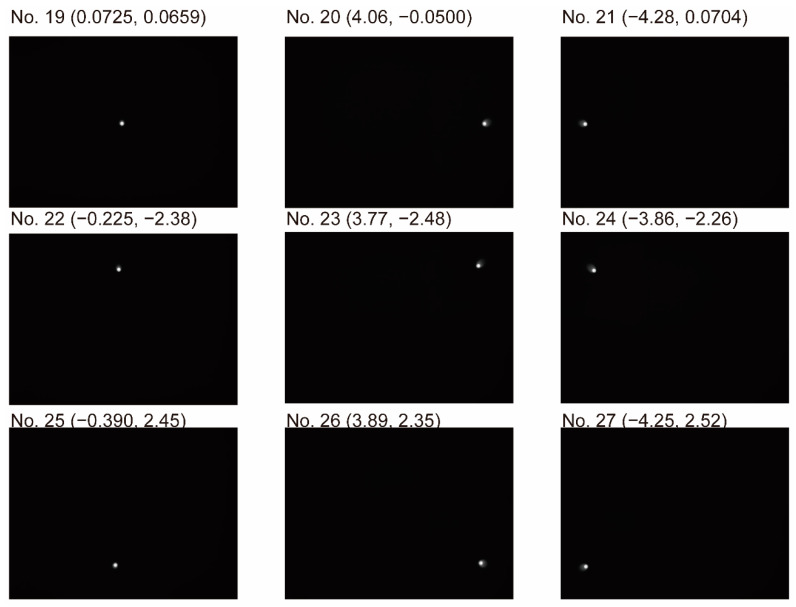
Captured thermal images of the light source at 2 m from the hybrid lens.

**Figure A6:**
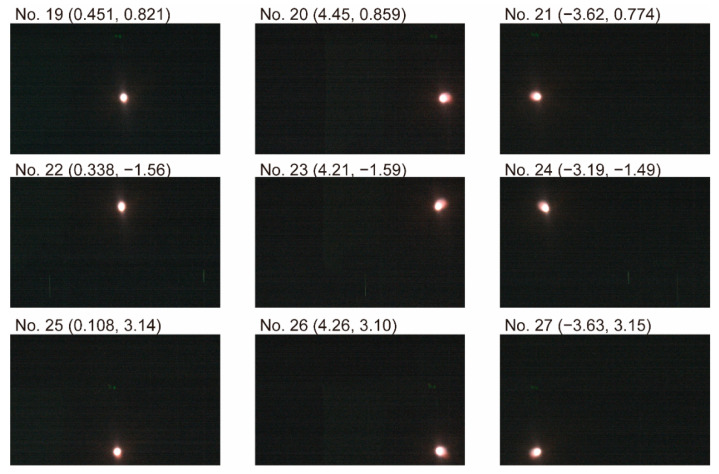
Captured color images of the light source at 2 m from the hybrid lens.

Figure 7 provides examples of thermal and color images of the light source. Figure A1, Figure A2, Figure A3, Figure A4, Figure A5 and Figure A6 in Appendix A show all 27 image pairs that were captured. The coordinates of the light source in each image were calculated to be those of the center of gravity of the pixels whose brightness’s were higher than a threshold value. The thresholds for the thermal and color images were set to 150 and 254, respectively. Figure 8 shows the nine positions of the captured light source at the thermal and color imagers. A total of 27 image pairs were captured, as described in Section 2.3.

### 3.2. Estimated Camera Parameters

The intrinsic parameters of the thermal and color cameras as well as the extrinsic parameters of the thermal camera were estimated using the 27 image pairs. The value of the objective function (Equation (3)) after estimation was 0.0237. The values of the objective functions for the thermal and color images (Equations (1) and (2) were 0.0401 and 0.0143, corresponding to 2.36 and 2.44 px, respectively. The errors represented in pixel units were balanced becauspixe of the weights $w^{\mathrm{col}}$ and $w^{\mathrm{th}}$, as mentioned in Section 2.4.

The estimated intrinsic parameters of the thermal camera were $f_{\mathrm{th},x}=51.5$ mm, $f_{\mathrm{th},y}=51.4$ mm, $c_{\mathrm{th},x}=0.00547$ mm, and $c_{\mathrm{th},y}=-0.788$ mm. The estimated intrinsic parameters of the color camera were $f_{\mathrm{col},x}=50.0$ mm, $f_{\mathrm{col},y}=49.9$ mm, $c_{\mathrm{col},x}=0.524$ mm, and $c_{\mathrm{col},y}=-0.00549$ mm. These estimated values agree well with the designed values of f=50 mm and c=0 mm. The result for the estimated focal length of the thermal camera indicated that the focal length of the outer silicon lens was slightly larger than the designed value. The misalignment of the principal points with respect to the design was less than 1 mm.

The estimated extrinsic parameters of the thermal camera were $q={\left[\begin{array}{c} \begin{array}{cccc} -7.46\times 10^{-5} & -5.25\times 10^{-3} & -1.82\times 10^{-3} & 0.9999846 \end{array} \end{array}\right]}^{T}$ and $t={\left[\begin{array}{ccc} -0.0343 & -0.111 & -4.08 \end{array}\right]}^{T}$. From the estimated quaternion q, the angle difference δ between the normal vectors of the thermal and color imagers was calculated to be $\delta =5.55\times 10^{-3}$ rad. The estimated translation vector t indicated that the translation along the directions parallel to the imager was small, whereas the translation along the normal direction of the imager, i.e., the optical axis direction, was relatively large. The small values of δ,$\;t_{x}$, and $t_{y}$ indicated that the inner and outer lenses of the hybrid lens were well aligned. The relatively large $t_{z}$ was assumed to be due to the relative displacement of the two imagers along each optical axis.

The other estimated parameters, which are the light source positions for the 27 image pairs, are shown in Table A1 in Appendix B.

In summary, the thermal and color imagers had a misalignment as small as $5.55\times 10^{-3}$ rad in rotation, a relatively small (1 mm) misalignment along the directions parallel to the imager, and a relatively large (4.08 mm) misalignment along the normal direction of the imager.

### 3.3. Estimated Mapping from Thermal to Color Images

The mapping from the thermal to the color images was estimated according to the equations in Section 2.5. Three sets of mapping parameters were estimated by capturing the light source at distances of 0.5 m (image pairs No. 1–9 in Figure A1 and Figure A2), 1 m (image pairs No. 10–18 in Figure A3 and Figure A4), and 2 m (image pairs No. 19–27 in Figure A5 and Figure A6), respectively. Using each estimated parameter set, the RMSEs between the coordinates in the color image and mapped thermal image (Equation (4)) of the light source at distances of 0.5, 1, and 2 m were calculated and are shown in Table 2. The RMSEs of the image pairs used for estimation were less than 0.00964 mm, corresponding to 0.567 and 1.65 pixels in the thermal and color images, respectively, and indicating that the estimations were successful. The maximum RMSE of 0.0264 mm corresponded to 1.6 and 4.5 px in the thermal and color images, respectively.

Table 3 lists the estimated parameters. All three parameter sets were found to be similar to each other. The rotation angles θ, lateral translations a, vertical translations b, and scaling factors s were approximately $1.4\times 10^{-2}$ rad, 0.54 mm, 0.79 mm, and 0.97, respectively.

**Figure 9:**
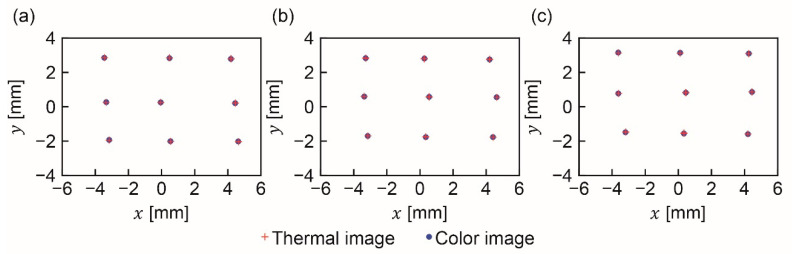
Positions of the light source at distances of (**a**) 0.5, (**b**) 1, and (**c**) 2 m in captured color images and mapped thermal images. The mapping was estimated using pairs of images of the light source at a distance of 1 m.

**Figure A7:**
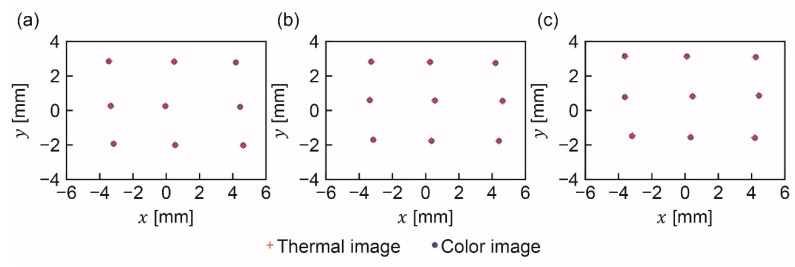
Positions of the light source at distances of (**a**) 0.5, (**b**) 1, and (**c**) 2 m on captured color images and mapped thermal images. The mapping was estimated using pairs of images of the light source at a distance of 0.5 m.

**Figure A8:**
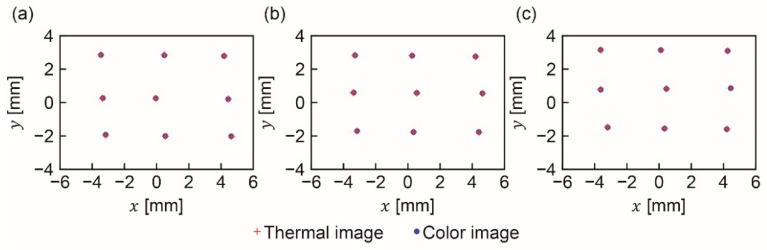
Positions of the light source at distances of (**a**) 0.5, (**b**) 1, and (**c**) 2 m on captured color images and mapped thermal images. The mapping was estimated using pairs of images of the light source at a distance of 2 m.

Figure 9 presents the mapping results estimated using pairs of images of the light source at a distance of 1 m, and Figure A7 and Figure A8 in Appendix C provide the results obtained using pairs of images at distances of 0.5 m and 2 m, respectively. As shown in these figures, the coordinates of the light source in the mapped thermal images are very similar to those in the captured color images.

## 4. Discussion

As can be seen in Figure 8, the positions of the light source in the thermal and color images are not identical. A probable reason for the discrepancy is misalignment of the optical elements: the outer and inner components of the hybrid lens, the beam splitter, and the thermal and color imagers. In general, even a well-tuned coaxial optical system can be slightly misaligned. Hence, it is important to create an index for evaluating coaxiality.

In this study, we proposed and verified two hypotheses to evaluate the coaxiality of a coaxial system. Hypothesis 1 was related to the intrinsic and extrinsic parameters of thermal and color cameras in 3D space. Hypothesis 2 pertained to mapping thermal images onto color images in 2D space. The parameters estimated in the process of verifying both hypotheses indicate correspondence between the thermal and color cameras; therefore, these hypotheses should be linked. The estimated rotation angle θ given in Section 3.3 is small ($\theta <1.5\times 10^{-2}$ rad), which is consistent with the estimated small angle difference δ between the two imagers of $5.55\times 10^{-3}$ rad in Section 3.2. The translation (a, b) from a thermal image to a color image was estimated to be (0.54, 0.79), as stated in Section 3.3, which is consistent with the difference between the estimated principal points, $\left(-sc_{\mathrm{th},x}+c_{\mathrm{col},x},\;-sc_{\mathrm{th},y}+c_{\mathrm{col},y}\right)=\left(0.52,\;0.76\right)$. The scaling factor s of 0.97 is consistent with the ratio of the estimated focal length of the inner lens to that of the outer lens: $\left(f_{\mathrm{col},x}+f_{\mathrm{col},y}\right)/\left(f_{\mathrm{th},x}+f_{\mathrm{th},y}\right)=50.0/51.4=0.97$.

There are two issues regarding the usage of a point light source, which was utilized in the experiment in this study. First, the accuracy of the light source position was not very good. The numbers of pixels whose brightness’s were higher than the thresholds in the color and thermal images were 1350 ± 210 and 65 ± 4 px, respectively. The light source position, which was calculated as the center of gravity of the pixels in each image, had an accuracy that was not better than several pixels. Therefore, the resulting RMSEs of 2.36 thermal pixels in Section 3.2 and 1.6 thermal pixels in Section 3.3 may be due to inaccuracies in the position calculation. As a second issue, a simple camera model without distortion was adopted in the analyses described in Section 2.4. In general, distortion can occur at the edges of images. When we calculated the RSME for each image using Equations (2) and (3), the RSMEs of the images in which the light source was placed near the corner, for example images 5–9 in Figure A1 and Figure A2, tended to be larger. However, the number of image pairs (27) was not sufficient to estimate the distortion parameters. These problems can be solved by using a type of calibration board that supports both thermal and color cameras, as described in the literature [5,7,8,17,20,21]. The method of coaxiality evaluation proposed in this report can also be adopted for images obtained using a calibration board. It should be noted that this report presents an index to evaluate coaxiality, rather than a calibration method.

The coaxial imaging systems reported both in the literature and this paper used a beam splitter aligned at ~45° with respect to the optical axis to separate visible and LWIR light. In this paper, a silicon plate was used as a cold mirror. Because LWIR light was refracted by the plate, as shown in Figure 3a, it can be assumed that the resolution of the thermal images is slightly diminished. However, it was also assumed that the resolution of color images was reduced in coaxial imaging systems using hot mirrors, due to the same effect. In either case, one of the images will be affected by the beam splitter in this way. Therefore, one should decide which optical system to use depending on the target application.

**Figure 10:**
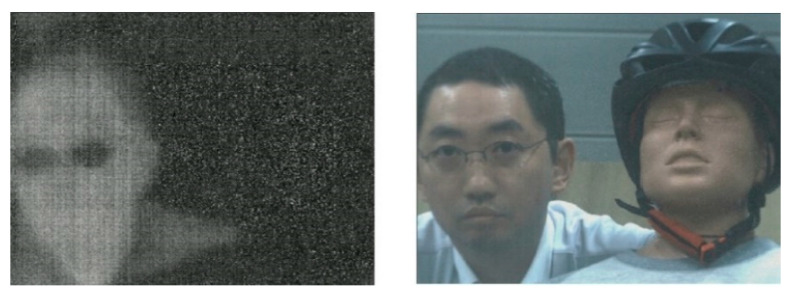
Thermal and visible images of a human and doll.

Since the proposed imaging system is as small as 140 mm × 140 mm, it can be used in numerous applications, including human detection, 3D thermal mapping, and noninvasive measurement in agriculture. Thermal and color images of a demonstration movie of a human wearing spectacles and a doll are shown in Figure 10. From the thermal image, one can identify that the object on the left is at a temperature exceeding room temperature and is wearing spectacles. As an issue to be solved in future research is the that observed signal to noise of thermal and color images captured by the proposed system is lower than those of images captured by independent color and thermal cameras. The quality can be improved by applying an anti-reflection coating to the outer silicon portion of the hybrid lens and the beam splitter.

## 5. Conclusions

This report presented a compact imaging system employing a concentric silicon–glass hybrid lens to capture thermal and color images of objects from the same point of view and with the same viewing angle. The spatial overlap of the optical axes of the thermal and color images was made possible by the use of the single hybrid lens. The viewing angle for both images was adjusted using similarly sized thermal and color imagers. We proposed two hypotheses regarding the coaxiality of the presented imaging system: first, the two imagers have the same camera parameters, and second, the mapping between the thermal and color images can be estimated using pairs of images of an object at a specific distance from the system. By verifying these two hypotheses, we experimentally demonstrated the coaxiality of the system. The proposed methods of evaluating coaxiality can be applied to other types of thermal and color coaxial imaging systems.

## Figures and Tables

**Table 1:** Specifications of thermal and color cameras.

	Thermal Camera [26]	Color Camera [27]
Manufacturer	Optris	The Imaging Source
Model	PI640	DFK 33UX174
Number of pixels	640 × 480	1920 × 1200
PC interface	USB 2.0	USB 3.0
Spectral range	7.5–13 μm	0.40–0.65 μm (with IR cut filter)
Frame rate	32 fps	54 fps (RGB24 ^2^)
Imager manufacturer and model	(not available)	Sony IMX174LQ
Pixel size	17 μm × 17 μm	5.86 μm × 5.86 μm
Imager size ^1^	10.88 mm × 8.16 mm	11.25 mm × 7.03 mm

^1^ The imager size was calculated by multiplying the number of pixels by the pixel size. ^2^ Each pixel is represented by 24 bits, each of which 8 bits are for R, G, and B.

**Table 2:** Root-mean-square errors (RMSEs) of Euclidean distances between the positions of the light sources in the color images and mapped thermal images. Each RMSE was calculated using nine image pairs.

Distance to the Light Source for the Images Under Evaluation	Distance to the Light Source for the Images Used for Estimation		
	0.5 m	1 m	2 m
0.5 m	0.00964 mm	0.0157 mm	0.0264 mm
1 m	0.0153 mm	0.00947 mm	0.0169 mm
2 m	0.0252 mm	0.0163 mm	0.00823 mm

**Table 3:** Estimated parameters for mapping thermal images to color images.

	Distance to the Light Source for the Images Used for Estimation		
	0.5 m	1 m	2 m
Rotation angle θ [rad]	0.0139	0.0147	0.0136
Lateral translation a [mm]	0.536	0.539	0.535
Vertical translation b [mm]	0.794	0.790	0.786
Scaling factor s	0.963	0.966	0.969

## Appendix A

Figure A1, Figure A2, Figure A3, Figure A4, Figure A5 and Figure A6 present color and thermal images of the light source at distances of 0.5, 1, and 2 m as well as the calculated coordinates on the corresponding imagers. The coordinates are expressed in millimeters for the reason explained in Section 2.4.

## Appendix B

Table A1 provides the entire list of the coordinates of the light source estimated as described in Section 3.3.

**Table A1:** Coordinates of the light source estimated as described in Section 3.3. All values are expressed using four significant figures.

Image Pair Number	Estimated Coordinates of Light Source			Image Pair Number	Estimated Coordinates of Light Source		
	X	Y	Z		X	Y	Z
1	−0.5268	2.543	500.0	15	−67.30	−34.07	1000
2	44.45	2.083	500.0	16	7.011	56.16	1000
3	−33.37	2.634	500.0	17	88.23	55.17	1000
4	5.370	−20.08	500.0	18	−65.58	56.59	1000
5	46.32	−20.27	500.0	19	17.94	32.89	2000
6	−31.63	−19.31	500.0	20	178.0	34.46	2000
7	4.760	28.35	500.0	21	−145.0	30.93	2000
8	41.88	27.90	500.0	22	13.36	−62.32	2000
9	−34.52	28.54	500.0	23	168.3	−63.94	2000
10	11.07	11.52	1000	24	−127.7	−59.48	2000
11	92.68	11.04	1000	25	4.243	125.7	2000
12	−67.30	11.95	1000	26	170.3	124.1	2000
13	5.259	−35.35	1000	27	−145.2	126.1	2000
14	84.1	−35.5	1000				

## Appendix C

Figure A7 and Figure A8 present the results of mapping a thermal image to a color image, estimated using the pairs of images of the light source at distances of 0.5 m and 2 m, respectively.
